# Probing the Underlying Principles of Perceived Immanent Accents Using a Modeling Approach

**DOI:** 10.3389/fpsyg.2019.01024

**Published:** 2019-06-07

**Authors:** Anders Friberg, Erica Bisesi, Anna Rita Addessi, Mario Baroni

**Affiliations:** ^1^Department of Speech, Music and Hearing, Electrical Engineering and Computer Science, KTH Royal Institute of Technology, Stockholm, Sweden; ^2^Laboratory “Perception and Memory”, Department of Neuroscience, Institut Pasteur, Paris, France; ^3^Department of Education Studies, University of Bologna, Bologna, Italy; ^4^Department of Arts, University of Bologna, Bologna, Italy

**Keywords:** immanent accent, music analysis, melody, modeling, machine learning

## Abstract

This article deals with the question of how the perception of the “immanent accents” can be predicted and modeled. By immanent accent we mean any musical event in the score that is related to important points in the musical structure (e.g., tactus positions, melodic peaks) and is therefore able to capture the attention of a listener. Our aim was to investigate the underlying principles of these accented notes by combining quantitative modeling, music analysis and experimental methods. A listening experiment was conducted where 30 participants indicated perceived accented notes for 60 melodies, vocal and instrumental, selected from Baroque, Romantic and Post-tonal styles. This produced a large and unique collection of perceptual data about the perceived immanent accents, organized by styles consisting of vocal and instrumental melodies within Western art music. The music analysis of the indicated accents provided a preliminary list of musical features that could be identified as possible reasons for the raters’ perception of the immanent accents. These features related to the score in different ways, e.g., repeated fragments, single notes, or overall structure. A modeling approach was used to quantify the influence of feature groups related to pitch contour, tempo, timing, simple phrasing, and meter. A set of 43 computational features was defined from the music analysis and previous studies and extracted from the score representation. The mean ratings of the participants were predicted using multiple linear regression and support vector regression. The latter method (using cross-validation) obtained the best result of about 66% explained variance (*r* = 0.81) across all melodies and for a selected group of raters. The independent contribution of each feature group was relatively high for pitch contour and timing (9.6 and 7.0%). There were also significant contributions from tempo (4.5%), simple phrasing (4.4%), and meter (3.9%). Interestingly, the independent contribution varied greatly across participants, implying different listener strategies, and also some variability across different styles. The large differences among listeners emphasize the importance of considering the individual listener’s perception in future research in music perception.

## Introduction

A large body of studies about the perception of melodies shows that certain notes “stick out” and are more important than others; thus, they are perceptually *accented* (e.g., Cooper and Meyer, 1960; Lerdahl and Jackendoff, 1983; Clarke, 1988; Drake and Palmer, 1993; Huron and Royal, 1996). These accented notes are more easily remembered, and thus may form the anchors for a perceptual representation of a melody (Jones et al., 1987; Monahan et al., 1987). In this way, they may provide temporal markers for forming a metrical grid (Large and Jones, 1999) or a tonal context. This role as a predecessor to more advanced concepts of music perception indicates that the formation of perceptual accents may be processed at a psychophysical, semiautomatic level (Müllensiefen et al., 2009). In this study, we try to further investigate the mechanism of these perceptually formed accents using a data-driven approach with a relatively large set consisting of a variety of different melodies. We will discuss previous work that is directly relevant for the present study. For a more detailed account of previous literature concerning accents in music, we refer to the comprehensive overview by Müllensiefen et al. (2009) and Bisesi et al. (2019).

Starting from a perceptual point of view, in this study we define an accent as an event that occurs when a note or sonority appears to be perceptually more important than the other notes. This can potentially be due to any kind of variation in any musical parameter, such as note duration, pitch, or dynamics. We divide such accents into “immanent” and “performed” accents as suggested by Parncutt (2003, p. 164): “Immanent accents are assumed to be apparent from the notated score; performed accents are effectively added to the score by a performer.” (see also Bisesi and Parncutt, 2011; Parncutt et al., 2013; Friberg and Bisesi, 2014; Bisesi et al., 2019). Thus, immanent accents can occur due to notated differences in the score regarding e.g., pitches, note values, rests, or chord structures. These perceived immanent accents are the focus of this study. Performed accents can occur, for instance, when the performer dynamically emphasizes a note or changes the duration or onset time. This is in our view a well-defined distinction, with the exception of the score instructions for the performance, in particular changes in dynamics (*f*, *p*, *sforzando*, *marcato*) or articulation (*legato*, *staccato*). Should these performance marks in the score be classified as performed or immanent accents? We chose to treat these performance marks as performed accents; thus, they were disregarded in this study. Indeed, from an experimental point of view, it is problematic to include them as score properties since there are no strict rules specifying how to translate a performance mark into physical values of time or sound level (Kosta et al., 2016, 2018). In order to clarify this point one more time, we conclude that our definition of immanent accents is perceptually originated due to the inherent properties of the score disregarding any performance variations or accent marks. This implies that these accents correspond to the perceptual emphasis formed when the music is played using a computer in a deadpan performance, i.e., when the score is directly translated into the corresponding sound without any expressive variation, using only note values and pitches.

What is the relation between immanent accents and performed accents? We assume that the most efficient communication from a performer to a listener occurs when the performance strategies (e.g., emphasis on one note) are used to further enhance the immanent accents that are inherent in the score. This idea was previously used as a general starting point for developing principles for expressive performance and implementing these principles into computational models (Friberg and Battel, 2002; Friberg et al., 2006). A similar situation occurs in speech, when the meaning of a sentence (to a large extent interpretable from the written text) is emphasized by means of speech accentuation involving, for example, pitch and duration variations (Carlson et al., 1989). In music, the way in which a performer may express immanent score content in her/his performance is influenced by a number of aspects, including perceived and felt emotion, or gestural and aesthetical intention (Friberg et al., 2006). Drake and Palmer (1993) confirmed the direct connection between immanent and performed accents, but also stated that there exists no simple one-to-one relation between the two domains, and that an interaction between different parameters should be taken into account.

Alternative taxonomies of accents have been suggested in previous studies. The basic principles are often similar, but the subdivision into different accent types is different from the definition of immanent vs. performed accent used here. For example, Lerdahl and Jackendoff (1983) describe the *phenomenal accent* as related to any variation in musical parameters such as local stresses, pitch, note duration or change in articulation, thus including both immanent and performed accents according to our definition.

Suggested underlying factors that may contribute to the perception of immanent accents (e.g., Monahan and Carterette, 1985; Parncutt, 1994) are melodic contour, relatively long notes, repetition of small melodic groups, perceived meter, perceived harmonic relations (i.e., chords perceived as dissonant or notes perceived as not belonging to the underlying harmony). In addition, there seems to exist an important contribution from the interaction among different basic parameters; thus, the accent is strengthened if several principles coincide (Jones and Boltz, 1989; Bisesi et al., 2019).

Another theory that discussed this topic from the perceptual point of view is the model of cue abstraction by Deliège (2001, 2007), who proposed the concept of cues as perceptual “prominent features” of music. This model, even if it does not coincide with the concept of accent, can be useful to better understand the cognitive process of perception of the musical accents during listening. According to Deliège, these cues are “prominent features” which are perceived and memorized by the listener during the listening process. Studies carried out by Deliège (2001, 2007) show how the listener, when faced with music that is not ruled by tonal structures, tends to identify certain qualities of sameness and difference which can easily be perceived and memorized, allowing the listener to abstract “cues” (or prominent features) which distinguish one part of the piece from another. These cues, which in tonal music are provided by the hierarchical harmonic, melodic and rhythmic structures, can also appear in non-tonal music, above all as elements linked to dynamics, duration, tempo and timbre.

In our previous experimental studies, we analyzed how the listeners (musicians and non-musicians) memorize the overall structure of post-tonal music during listening in real time (Addessi and Caterina, 2005; Addessi, 2010). We observed that the perception of “prominent features” allowed the listeners to perceive several points of segmentations, to divide the piece into the main sections and consequently, to memorize the overall structure of the musical piece. It was also found that there was a correlation between the memorization of the overall structure and the perception of tension and relaxation (Addessi and Caterina, 2000). We listed several musical categories trying to describe the musical features of the perceived “cues”: variation in intensity, timbre variation, acceleration/deceleration or change in rhythm, thickening or thinning of the sound, introduction, repetition, elements concluding or suspending, and pause. The musicians in comparison with non-musicians indicated the category “timbre variation” and “introduction, repetition” as the most important to explain segmentation; on the other hand, the category “variation in intensity” was indicated by non-musicians. However, in these studies, the participants were asked to indicate the points of segmentation and division in sections, and not the accents.

Previous computational models of accents are mostly based on local context principles for rhythm, pitch, meter, and harmony (e.g., Thomassen, 1982; Parncutt, 1994; Müllensiefen et al., 2009; Friberg and Bisesi, 2014; Bisesi et al., 2019). Thomassen (1982) focused on the melodic contour and formulated a model based on three-note motives with the same note values and different pitch patterns. The accent weights of the model were distributed among the second and third note depending on the direction of the two intervals. The weight values were determined from experimental data using a “method of controlled anticipation” in which three-note sequences containing the pitch variation were rated in terms of “regularity” in relation to an induced metrical grid. A resulting correlation of *r* = 0.76 was obtained for the comparison of experimental data versus model predictions for four-note motives.

A quantitative model of pulse salience and metrical accent in musical rhythms which depends on the inter-onset intervals between consequent notes was presented by Parncutt (1994). His model was then translated into two performance rules: (a) local *ritardandi* in the vicinity of metrical accents and (b) non-metrical events are shortened. Following this approach, one can model timing variation in a way that is mathematically simpler, intrinsically tempo dependent, non-local (i.e., depending not only on immediately preceding and following notes, but on all notes in a time span of, say, 2 to 8 s), and more closely related to fundamental perceptual parameters in rhythm perception, like pulse salience and metrical accents themselves.

Parncutt, Bisesi, and Friberg formulated a preliminary computational model of metrical and melodic contour accents based on intuitive principles (Bisesi and Parncutt, 2011; Parncutt et al., 2013). By assuming musicological principles (e.g., pertaining to hypermeter, melodic climax) and formulating algorithmic predictions for accents’ positions and salience that were further evaluated either informally or by comparing predictions with data, these studies adopted a top-down approach. Such a model was subsequently applied to different musical styles (Friberg and Bisesi, 2014). In a recent study, the same authors presented a refinement of their model in which previous intuitive principles were further improved, and a new model for harmonic accent including both harmonic dissonance and harmonic surprise was introduced. That model was then compared with and improved using data from two experiments, in which musicians and music theorists were asked to mark the different accents on the score (Bisesi et al., 2019). In that study, a separate model for each of the three accent categories (melodic contour, metrical, and harmonic accents) was developed and evaluated. In this study, we chose instead to try a different approach and to mix all the accent categories together in a novel listening experiment. We also added a large number of new computational features partly derived from other studies and partly from the music analysis presented in this study.

Müllensiefen et al. (2009) formulated a general model of melodic accent using an exploratory approach in which a set of 38 mostly binary features (rules) were introduced, starting from and extending previous research including, for example, the model by Thomassen (1982). Accent ratings by 29 participants were collected from 15 pop melodies presented both as audio excepts and midi renditions. Logistic regression and regression trees were used to evaluate the influence of the features on the perceived accents and to formulate a simpler model using an optimal subset of features.

In contrast to Bisesi et al. (2019), the method used in this study is to a large extent data-driven and uses a bottom-up approach similar to that of Müllensiefen et al. (2009). First, we define a large set of features following previous research (e.g., Friberg et al., 1998; Müllensiefen et al., 2009; Bisesi et al., 2019). Then, we let the model learn from the data and combine the different principles automatically using data prediction methods [multiple linear regression (MLR), support vector regression (SVR)]. We also manually analyzed some of the participants’ accent ratings in relation to the score in order to further understand their strategies. From the final modeling, we can identify those basic principles that are important for the listeners. Our approach differs from Müllensiefen et al. (2009) in several respects. For example, we are using an extended set of 60 melodies taken from different styles of Western art music (instead of 15 pop melodies) and the prediction and analysis methods including the evaluation of feature importance are different.

This paper is structured into four main sections. *Music corpus*. A corpus of 60 melodies was selected from Baroque, Romantic, and Post-tonal repertoire: 30 instrumental melodies (10 for each style) and 30 vocal melodies (10 for each style). *Rating experiment*. Perceptual data were collected involving 30 listeners, mainly amateur musicians, who were asked to indicate the most important notes while listening to each melody. *Music analysis*. A music analysis of the melodies was performed in order to identify the musical properties that could be responsible for the accent marks obtained in the listening experiment. *Computational modeling.* A set of 43 features was defined and models were formulated that predicted the collected perceptual data from the extracted features.

## Music Corpus

In our study, we chose a differentiated repertory of Western art music, spanning from baroque to modern styles, as we wanted to have a reasonable variation of musical content. More specifically, the selection criteria included the three different styles “Baroque,” “Romantic,” and “Post-tonal,” both vocal and instrumental music, and a variety of different composers. These three styles mainly distinguish melodies of the 17th and 18th centuries from melodies belonging to the first eight decades of the 19th century and the transitions from romantic to modern styles (for example Brahms, Mahler etc.). The third style, “Post-tonal,” refers to the universe of melodies of the 20th century. However, we excluded from the third category oral or primarily non-notated music (e.g., the majority of jazz, popular, or computer-based music); thus, we selected only notated melodies within the Western art music tradition. The total number of melodies was 60, equally divided among the three styles (20 in each) and then further divided between vocal and instrumental (10 in each). We limited the selection to 60 examples since we estimated this to be the highest possible number of melodies that could be rated by the participants in two sessions. The complete list is provided in Supplementary Table S1. Since we are using machine learning methods for the modeling, it is important to have a large database. From a musicological point of view, we are aware that this is a rather modest selection that cannot be considered a representative sample of these three styles. However, we think that it can represent a reasonable sample and provide a rather large melodic variation in relation to perceived accents.

## Recording

For our experimental purpose, only the melodies were extracted from the scores, disregarding any other parts such as accompaniment. Following our definition of perceived immanent accents, the melodies were rendered using only nominal pitch and note values, disregarding any additional score information, such as *crescendo*, *sforzando*, and *accelerando* marks. The melodies were first coded in *mscz* format with the free notation software MuseScore 2.1, and then exported as *xml* and converted into the Director Musices (DM) (Friberg et al., 2006) *mus* format by means of the Humdrum Toolkit (Huron, 2002) (following the sequence: *xml*, *krn*, *mus*). The *mus* files were then imported in DM and checked for compatibility. Performance tempi were extracted from examples provided by professional performances of the melodies. The duration of each extract lasted approximately 30 s. A midi output was also created as an input for the experimental interface (see section “Procedure”).

The melodies were played by the computer using DM version 3.1.1, without any performance variation on a midi-controlled Disklavier Yamaha C3pro grand piano, and then recorded in audio format. Recordings were made by means of professional recording equipment (RME Fireface 800 audio card, two Brüel & Kjær 4003 microphones mounted 20 cm above the strings, Apple MacBook Pro computer using Audacity 2.1.1 software). To cope with calibration inhomogeneity in the Disklavier and due to the room acoustics in relation to microphone positions, we adjusted the dynamics of each tone of the keyboard by ear, until we obtained uniform perceived dynamics along the whole keyboard range (verification was made by looking at the spectrogram representation in Audacity). The new values of dynamics were then coded as small deviations in sound level for each note in DM and applied to the melodies.

## Listening Experiment

### Method

#### Participants

Thirty musicians voluntarily participated in the study (17 males and 13 females; age, mean = 44.15, *SD* = 16.0, min = 20, max = 73 yr). About half of them were recruited from the KTH amateur orchestra in Stockholm and half from amateur choirs in Stockholm. Participants indicated their expertise at the beginning of the task according to the following point scale from 1 to 5: 1 = non-musician and non-listener; 2 = non-musician and listener; 3 = amateur musician; 4 = musician with a degree in music; 5 = professional musician. The average self-reported expertise was 3.4 with a standard deviation of 0.77. They were originally from Sweden (22), Germany (2), France (2), Italy (1), Spain (1), Russia (1), and Greece (1). Most of them were amateur performers (25 classical; 5 jazz), but in a few cases, they stated that they were professional musicians (1 classical and 3 jazz). Most participants reported that they played one or more instruments (piano or other keyboards, violin, viola, guitar, flute, clarinet, oboe, bassoon, saxophone, percussions, drums, jazz band, or voice), or sang in a choir. A few of the participants were music teachers, two were conductors, another two were composers, and one was also a dancer. The number of participants was chosen from the experience of our previous studies about perceptual features, in which 20 participants was considered to be enough to make a mean value estimation (Friberg et al., 2014) and from previous studies about accents (Bisesi et al., 2019) which showed a large variability in the participants’ responses. Thus, 30 participants were considered an acceptable number to make a mean value estimation and to study individual differences. The experiment took place in the KTH Multi Studio in Stockholm.

#### Procedure

**Figure 1:**
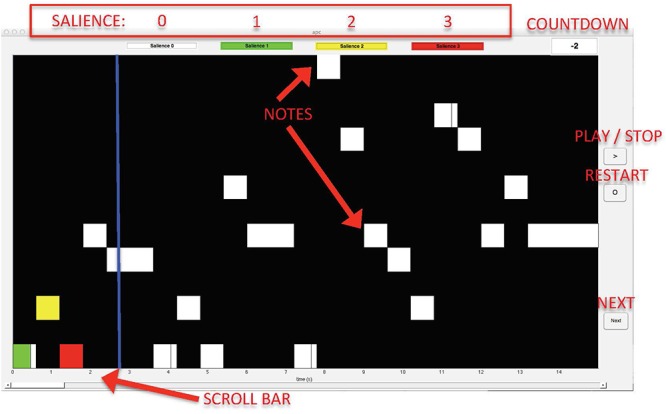
Graphic interface used in the experiment.

To perform the task, we specifically developed a computer interface using Matlab R2017a, where the audio was aligned to a corresponding midi piano roll display. A blue bar followed the notes, which were displayed as white rectangles on a black background (see Figure 1). This interface was introduced to reduce the bias associated with the score, so the only information about the pieces that was provided to the participants regarded the physical content of the signal [i.e., note onset and duration (on the *x*-axis) and pitch (on the *y*-axis)]. Our interface was provided with a scroll-bar and buttons (play, stop, restart, and next), enabling participants to listen to the melodies as many times as they wanted, as well as to stop the music at any time and/or listen to small portions of the piece, and move to the next piece. As input, it required both midi and audio versions of each melody.

For each melody, participants were asked to indicate the most important notes according to their intuitive subjective perception, and to provide an estimation of the degree of *importance*^1^ of each note (or *salience*) on a point-colored scale from 1 to 3. To select a note, they clicked inside the corresponding white rectangle on the interface. To rate the salience, participants “colored” the boxes by clicking inside for one or more times according to the following loop-scale: 0 clicks, white = no importance; 1 click, green = just a little important; 2 clicks, yellow = intermediately important; 3 clicks, red = very important; 4 clicks, white = no importance, i.e., unselecting a previously selected event; etc. The melodies were presented in random order. Before moving to the next melody, a pop-up window appeared asking the participant to indicate whether the piece was known or not.

At the beginning of the experiment, participants signed a consent form informing them about the purpose and content of the study, ensuring that the procedure did not involve any discomfort or risk, and guaranteeing freedom to withdraw at any time and anonymity. Then the experimenter distributed and read instructions for the graphical interface. Before starting with the experiment, participants were provided with two familiar practice trials (*Happy birthday to you* and *Santa Lucia*) to get used to the task and were requested to fill in a form concerning some personal data (nickname, nationality, age, gender, expertise, and location). At the end of the task, they received a questionnaire concerning their education, music preferences, familiarity with the selected music styles, and strategies adopted for selection. All the participants declared to have fully understood the task.

The entire task was performed in two rounds separated by a couple of weeks or months. Participants were divided into two groups, each group receiving a different set of 30 melodies per round. Each round lasted around 120 min.

The final dataset consisted of 30 salience values (one from each participant) from 0 to 3 for each note of all melodies.

### Results

#### Agreement and Overall Measure of Ratings

**Figure 2:**
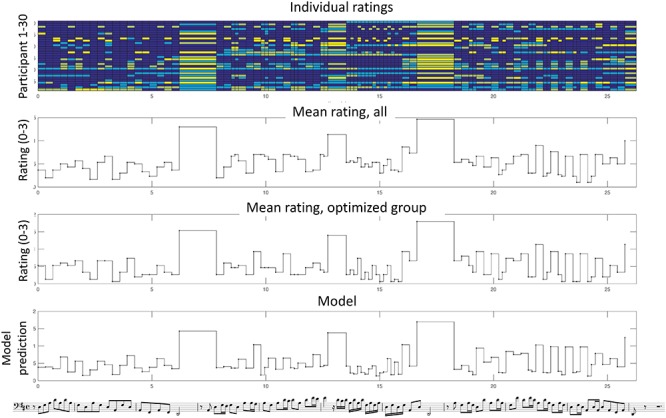
Individual and mean ratings for melody no. 1, *Toccata* for solo cello by Vitali. In the upper graph, the response of each participant (1–30) is color-coded according to the accent level (in color scale: 0, dark blue; 1, light blue, 2, dark yellow, 3, bright yellow). In the second and third graphs the mean ratings across all or across the optimized group are shown. The fourth graph shows the model results using the optimized group.

An important purpose of the data collection was to find a representative overall measure of the accent ratings for each note across all raters or across a selection of raters. This overall measure was then used for predicting accents’ positions and saliences in the model below. In this study, averages across participants were used as the overall measure. This resulted in a continuous measure in which most notes had a non-zero accent value (see Figure 2). Averaging across participants could be considered standard procedure in listening experiments. However, it does not always represent the majority of the listeners since outliers may have a large impact. An alternative method that was tried was to consider only those notes that had *m* number of raters who marked that note with an accent mark. Different values of *m* were tested, and the final overall accent was computed as the average across the remaining marks (see also Bisesi et al., 2019). In this case, the basic average measure was found to be more consistent than the selection of notes in a manual evaluation of the different methods. This was done by inspecting the outcome of the averaging methods for a selected number of pieces.

To estimate the reliability of the computed average of the collected perceptual data, we used Cronbach’s alpha (CA). CA is the same measure as the intra-class correlation ICC(C, k), case 2 (for the definition, see McGraw and Wong, 1996). To estimate the variance among participants, we used the average pair-wise Pearson’s correlation across all participants. The resulting values are shown in Table 1, for all melodies and also for each melodic group. As seen in the table, CA was 0.836 for all the melodies, and the mean pair-wise correlation was 0.157. The Cronbach alpha value indicates that the average across participants is a relevant approximation that can be used in the computational model. The pair-wise correlations indicate, however, that there is a large individual variation. Among the three styles, the highest value was obtained for the post-tonal melody group.

**Table 1:** Mean pair-wise correlations and Cronbach’s alpha (CA) for all participants and the different styles.

Melodies	Number of notes (cases)	Cronbach’s alpha	Mean pairwise correlation
All	4204	0.836	0.157
Baroque	1806	0.824	0.146
Romantic	1148	0.822	0.143
Post-tonal	1250	0.855	0.181
Instrumental	2635	0.836	0.157
Vocal	1569	0.827	0.150

For each melody, CA ranged from 0.677 to 0.936. For each pair of participants, the range of the pairwise correlation was -0.06 to 0.52. Thus, some participants had nothing in common, while others showed a relatively large agreement.

Given the rather low agreement in the pairwise correlations, we decided to investigate if there was a smaller group of participants that agreed to a higher extent with each other. Thus, in order to improve the estimation of the mean, we used an iterative method for selecting an *optimized group* of participants that would maximize CA. Starting with all the participants, they were omitted one by one, and a new CA value was calculated for each omission. The participant whose omission caused the highest increase in the CA was removed from the group and the procedure was repeated until the CA value no longer increased (see also Elowsson and Friberg, 2017). This resulted in a group of 15 participants with a CA value of 0.861 and a mean pair-wise correlation of 0.303. Thus, the mean correlation was almost twice as high in comparison with the whole group. In the subsequent analysis, we will report the results for both this optimized group and all the participants.

There may be several possible reasons for the rather small agreement among the participants. It may be related to differences in the strategies for selecting the “accented” notes, and also ascribed to random components due to the rather difficult task, as reported in the questionnaire below. In order to estimate the consistency among the raters, two melodies were repeated between the first and second session for a limited number of nine participants. The average correlation between the first and second rating of each of these two melodies was *r* = 0.50, ranging from 0.0 to 0.83. This large range indicates that the consistency among the raters varied substantially.

An illustration of the individual ratings and averages is shown in Figure 2 for an example from the instrumental Baroque group. As seen in the Figure, there is a substantial variability among the participants in the upper graph, which reveals a certain inconsistency possibly due to different strategies. However, when all the responses are averaged (both across all and for the optimized group), a more consistent pattern emerges in which several principles can be observed. For example, there is a strong accent on the three relatively long notes at phrase boundaries. In the 1st phrase, melodic peaks tend to be relatively more accented. In the 2nd phrase, the accents follow to a certain extent the rhythmic grouping of three or two notes.

#### Questionnaire

The participants’ backgrounds were further examined through a detailed questionnaire after the listening experiment concerning education, music preferences, familiarity with the selected music styles, and strategies used for the selection of the notes (see also Supplementary Material, Section 2).

The first part of the questionnaire concerned the degree of expertise. It contained questions about musical degree (if any), type of education (whether self-taught, or having received a regular school education, or having studied in a music school, in a Conservatory, or in a high-level Music Academy), specific knowledge of music theory, type of musical activity, and number of hours per week spent practicing and/or listening to music. These items were coded into a point scale from 1 to 5 and averaged, also including the participants’ own statements about their level of expertise that were provided at the beginning of the task (see section “Participants”). The resulting values for all participants are shown in Table 8, column 2. The distribution of expertise among the participants was rather evenly distributed in the range from 2 to 5, with most participants in the range of 2 to 4, indicating that the majority (20) were intermediate-level amateur musicians. The preferred genres of music were investigated as well, suggesting that in total 24 participants liked the proposed repertoire. The other parts of the questionnaire are reported in Supplementary Material. Although interesting, they were not used explicitly in the subsequent analysis.

## Music Analysis

We carried out a music analysis of the melodies with the aim of describing the musical features of the “most important notes” as indicated by the participants. Our purpose was not to describe general psychological principles, but rather to identify a preliminary set of musical features of perceived accents, starting from the accents indicated by the raters, which could be integrated in the computational features used in the subsequent modeling. The music analysis was carried out by two of the authors, musicologists who are experts in score and auditive music analysis. In the following, we will introduce the method, the preliminary list of musical features, and some results of the analysis of selected melodies.

### Method

We adopted a bottom-up method, moving from the answers of the listeners to a list of musical features which describe the points where the listeners indicated the accents. Our analysis was carried out in two steps: in the first step we identified a first set of musical features of perceived accents starting from the analysis of the answers of some of the raters; in the second step we analyzed a number of selected melodies testing the consistency of the list of musical features. We adopted a spiral method in which the results of step two were used to refine the results of step one, and vice-versa. In this sense, the result of the musical analysis is the first attempt to produce a list of musical features and it represents a work in progress and not a general psychological model. Specifically, the 1st and 2nd steps included the following substeps:

1st step: defining a preliminary list of musical features of perceived accents

(1a) The first phase consisted of the analysis of a number of responses by Rater 1. Accent by accent we made musical hypotheses on the reasons why this participant chose those accents. In this phase we analyzed the answers of Rater 1 in 9 melodies (1, 2, 3, 11, 12, 13, 21, 22, and 23).
(1b) Starting from the results of the analysis of answers of Rater 1, we analyzed the answers of other six raters and compared the different results.
(1c) Finally, we formulated a first draft of musical features which attempts to describe the musical characteristics of the most important notes indicated by the raters.

2nd step: music analysis of the corpus using the preliminary set of musical features

(2a) Analysis of the answers of selected raters. In this case, the focus of the analysis was on the individual strategies of the raters.
(2b) Analysis of selected melodies using the results of the “strong” accents with all participants (means). In this case, the focus of the analysis was the common strategies used by all participants (see the result sections below).
(2c) The results of 2a and 2b were used to refine the set of musical features (1c). For example, during the analysis of selected melodies 31, 36, and 41 (Sauli, Purcell, and Beethoven), it was observed that the perceived accents also depended on the overall structure (OS) of the melodies, and consequently the 3rd category—OS (see Table 2)—was added to the list of musical features.

**Table 2:** Preliminary list of musical features of perceived accents.

**1. FRAGMENTS**	**2. SINGLE NOTES**
**RF – Repeated Fragments:** Short melodic fragments which are repeated during the piece.	**SB – Strong Beats:** Notes placed in a metrical strong position; sb – strong beats in division/subdivision levels.
**AR – Arpeggio notes:** A fragment of ascending or descending arpeggio notes.	**WB – Weak Beats:** Notes placed in a metrical weak position; wb – weak beats in division/subdivision levels.
**BE – Notes on main beats:** Notes interconnected but not adjacent. They are metrically connected (they depend on meter) but they do not form a melody.	**S – Syncope:** Notes placed on a syncope.
	**A – Anacrusis:** Notes placed on an anacrusis.
	**LG – Long notes:** Notes longer than the previous and/or the following ones.
	**HAR:** Important harmonic function (modulation, dissonance, etc.).
	**T:** Tonal function (tonic, dominant, subdominant).
	**C:** end of cadence.
	**CL – Climax:** Notes which conclude an ascending or descending fragment; the highest or the lowest pitch of the fragment.
**3. OVERALL STRUCTURE OS –Overall Structure**: Note(s) that marks the division of the piece into main parts.	**LN – Last note:** The last note of a melodic fragment.
**T/R – Tension/Relaxation**: Note(s) that affects the perception of tension and relaxation.	**FN – First note:** The first note of a melodic fragment.
	**Ch – Change:** Notes which introduce a change or strong variation (in register, rhythm, tonality, etc.).
	**EM – Embellishment:** Notes placed on an embellishment.
	**UA – Uncertain accents:** Notes to which none of the previous categories could be applied.
	**TotAc:** Total number of accented single notes.
	**NA:** Total number of not accented single notes.

### Preliminary List of Musical Features of Perceived Accents

Table 2 shows a synthesis of the musical features that resulted from steps 1 and 2. This list is based on a selected number of raters and melodies and should not be considered definitive, but rather as a work in progress.

**Figure 3:**
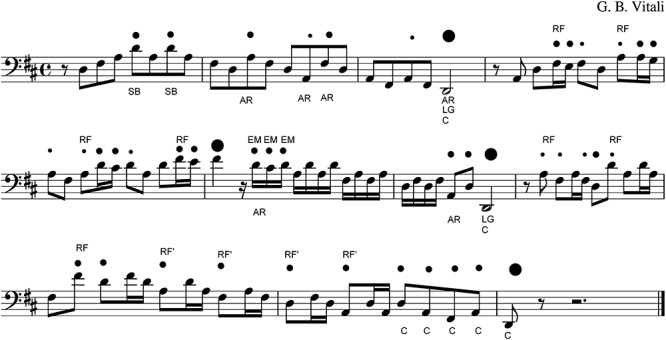
An example of the analysis of different accent types as indicated by the participants in melody no. 1 by Vitali.

**Figure 4:**
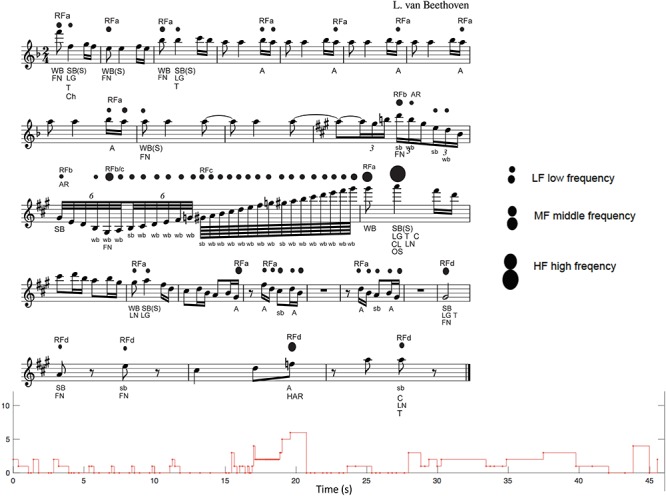
The analysis of strong accents by all participants in melody no. 41 by Beethoven. The figure shows the score and the graph with the mean of the strong accents indicated by all listeners. The score shows the strong accents (indicated by the dots) and the labels that describe the music features. The size of the dots indicates the frequency of answers: Low Frequency (LF), when the strong accent is indicated by few raters; MF (Medium Frequency), when the strong accent is indicated by a medium number of raters; and HF (High Frequency), when the strong accent is indicated by a high number of raters.

Three main categories of musical features were found:

1. *Fragments of notes*, which include RF = short melodic fragments, which are repeated during the piece (see the musical examples in Figure 3, 4), AR = fragment with ascending or descending arpeggio notes (AR; see Figure 3), and BE = notes which are metrically interconnected, even if they do not form a melody.

2. *Single notes*, which have different properties of salience (see Figure 4).
3. *Overall structure*, which includes the note(s) that segments the piece into main parts and the note(s) that creates tension and relaxation (see Figure 4).

Some examples of musical features of the accents indicated by the listeners in melody no. 1 by Vitali are shown in Figure 3. In the Figure, the accents indicated by the listeners are represented by means of the black dots: the little dots indicate a low frequency of answers (LF), which means that a low number of participants indicated the accent; the medium dots indicate the accent which received a middle frequency of answers (MF); and the bigger dots indicate the accents which received the higher frequency of answers (HF). Furthermore, several symbols indicate the feature of the accents, as described in Table 2. For example: in bar 1, we have 2 accents with middle frequency (MF) on the strong beat (SB). In bar 2 there are two accents with middle frequency (MF), which are on the first note of the descending arpeggios AR, and one accent with low frequency, which is on the last note of the arpeggio. In bar 3, there is a low frequency (LF) accent on the first note of the arpeggio and a high frequency (HF) accent on the last note of the arpeggio, which is a long note (LG) at the end of cadence (C). In bars 4–6 there is a repeated fragment (RF), which is repeated four times, finishing on the first note of bar 6, which is also a long note (LG) with HF of answers. In bar 6 there are then three accents (MF) on three consecutive short notes, like an embellishment (EM). In bar 7 there are two medium accents (MF) and one HF accent on cadence (C) and long note (LG). In bars 8–9 the RF appears once again and is repeated three times. In bars 9–10 a shorter variant of the same RF (RF’) is repeated four times. In the second part of bar 10, four medium accents (MF) are indicated on the cadence notes (C), and finally, in bar 11, the HF accent is indicated on the end note of the cadence (C).

With SB and weak beat (WB) we indicate the stressed and unstressed beats. We follow the general rule that the first downbeat of the bar is always going to be strong, while the following beats are weak. In 4/4 time the third beat is also considered strong. However, for syncopation (for example when there is a rest on the first downbeat of the bar), it turns upside down: strong means weak. In this case we indicate the syncopation by an S and indicate with WB the unstressed first beat. The subdivision levels (like eight-notes in 4/4 time) that fall between the pulse beats are also called weak. With anacrusis (A) we indicate “one or more note preceding the first metrically SB of a phrase; upbeat, pickup” (Randel, 2003, p. 42). This happens also when the anacrusis ends on a rest, as in measure 23, Figure 4 (see Cooper and Meyer, 1960, pp. 172–173).

### Results of Music Analysis

In the following, we will introduce the results of the analysis of the accents indicated by individual raters and the analysis of the “strong” accents indicated by all raters.

#### Results of Music Analysis of the Accents, With Individual Raters

We analyzed the accents provided by Rater 1 for 18 melodies and those provided by Raters 2–6 for three instrumental melodies and for three vocal melodies for each of them. The total number of analyzed melodies was 48 (24 instrumental and 24 vocal melodies). Melodies 1–8 pertain to the Baroque group, those from 11 to 18 to the Romantic group, and those from 21 to 28 to the Post-tonal group (see Supplementary Table S1). On the basis of the raters’ responses and taking into account that the total number of the notes indicated by this group of raters is 696, the percentages of the different features (excluding decimals) are shown in Table 3.

**Table 3:** Results of the music analysis of individual raters for the different types of accents.

Abbreviation	Explanation	Occurrence (%)
RF	Notes inside repeated fragments	29
UA	Accents with uncertain categorization	19
AR	Arpeggio notes in unidirectional fragments	13
C	Notes included in cadences	12
BE	Regular sequences of main beats	12
SB	Singular strong beats	7
LG	Isolated long notes	6
CL	Climax notes	3

We have a few comments on Table 3:

– In “RFs” and “AR” the accents are never indicated over all the notes, but only on some of them. Due to their number and position, we did not consider such accents as “singular,” but rather as belonging to the entire fragment. For an example, see Figure 3.
– For the category “Uncertain accents” (UA), we observed that they are particularly frequent in post-tonal style (Post-tonal = 10%; Romantic = 4%; Baroque = 1,6%), where the absence of tonal features removed the main clues for the presence of accents. In such cases, it may be that the raters indicated the presence of accents having poor and often not consistent orientations.
– In tonal examples, cadential movements are normally concluded by “Long notes” (LG).
– There is a difference between BE and SB. The former is a metrical phenomenon (regular accents on sequences of SBs, or even simply on succeeding beats); the latter is an isolated SB combined with other conditions (e.g., an ascending interval).

The different melodic styles produced evident differences among the kinds of accents. For example, the repetitive and chordal structure of Vitali’s piece (melody no. 1) produced dominant choices of RF and AR. Carissimi’s melody (no. 6) is less arpeggic in contour but is characterized by a continuously repeated rhythmic group. The French examples (melodies no. 3 by Couperin and no. 7 by Rameau) give prominence to EMs. In the Romantic style, more complex, personal, and irregular structures are present: this gives emphasis to more problematic choices, even to more “uncertain” categorizations of accents (UA). Other accent indications were variously shared by the different melodies without particular dominances. In the post-tonal style (e.g., melodies 21–28) there is an evident difference between melodies composed in the first 50 years of the 20th century (e.g., melodies no. 23 by Weill, no. 26 by Stravinsky, and no. 28 by Szymanowski) and melodies written in the second half of the century (e.g., melodies no. 21 by Maderna, no. 24 by Scelsi, and no. 27 by Berio). The former preserve more traditional (and often pre-romantic) accents (governed by metrical regularity); in the latter, the only recognizable accent (indicated by the majority of the raters) was often simply a longer duration of some notes.

#### Results of Music Analysis of the “Strong” Accents, With All Participants (Mean)

Another set of analyses was carried out on the “strong accents” indicated by all participants (mean). The aim was to find some common strategies followed by all participants. In this section, we will introduce the results of the analysis of the strong accents for three melodies: no. 41 by Beethoven, no. 31 by Sauli, and no. 36 by Purcell.

##### Melody No. 41 by Beethoven

As seen in Figure 4, there are four types of RF (RFs)—RFa, RFb, RFc, and RFd—for a total of 22 RFs. The most frequent RF is of type “a” (13 occurrences, bars 1–8, 13–17, and 19), which is characterized by the syncopation (S) with an anacrusis (A) in sixteenth notes, which are often accented, even if with LF, (see figure caption). The RFb (3 occurrences, bars 11–12) is characterized by descending sixteenth note triplets or thirty-second note sextuplets, and *arpeggiato*, excepting the third one, which presents the sextuplets but with ascending scale, like the following RFc. For this reason, we marked it as RFb/c on the score (see Figure 4, bar 12). The RFc is characterized by a faster ascending unidirectional scale of sixty-fourth notes, which leads to the climax (CL) note with the highest frequency of answers (HF) and highest pitch (bar 13), and once again to the RFa type with syncopation. RFc is present only once in the second part of measure 12, and it is interesting to observe that all 16 sixty-fourth notes in the scale are accented, although with a LF of answers. The RFd (5 occurrences, bars 21–24) is completely different from the previous fragments and is characterized by a single long note or a single note with a rest, which gives rise to a light and rarefied texture, leading to the conclusion of the piece. It is interesting to observe the musical features of the note with the higher number of answers (HF, bar 13): it is a long note (LG), on the SB of a syncopation (S), climax note (CL), the highest note of the piece, with tonally important function (T), in a cadence (C), the last note of a melodic fragment (LN), and it is included in the most frequent type of RF, RFa. It is interesting to observe that this note occurs at the maximum point of tension (T/R), and divides the piece into two parts, leading to the repetition of the initial RFa followed by the RFd, which leads to the conclusion and relaxation. In conclusion, the note with the higher frequency of answers (HF) gives rise to the perceived OS of the piece. Furthermore, we can also observe that in this melody there are more accented notes on WBs (*WB* = 30) than on SBs (*SB* = 16). For example, an accent is indicated on the anacrusis (A) in bar 23, in this case evidently due to an unexpected modulation (HAR). There are five accented long notes (LG), on strong positions and with important tonal function (T).

##### Melody No. 31 by Sauli

In this melody (score provided in the Supplementary Material, Supplementary Figure S2), it was possible to individuate three types of RF, characterized by the alternation of unidirectional scale fragments and RF with neighbor notes repeated 12 times, for a total of 14 RFs. The two notes with the higher number of answers (HF) were the highest notes of the song, in climax position (CL), on the SB, and with important tonal function (T). They are at the end (LN) and at the beginning of a melodic fragment (FN). The two HF notes divide the piece into three parts: the first part is characterized by unidirectional ascending fragments, the second part by both an ascending and a descending melody, and finally the third part with a unidirectional descending trend, moving from the sharpest note toward the conclusion. Therefore, again, the HF notes outline the macroform of the piece (OS).

##### Melody No. 36: Purcell

The melody no. 36 by Purcell contains many RFs and two EMs (score provided in the Supplementary Material, Supplementary Figure S3). This song is very slow and almost all the notes are accentuated (29 vs. 36). There are three types of RF that are repeated several times. It is interesting to observe that most of the accents are on metrically weak positions (WB) and that the accents on the metrically strong positions (SB) have a LF of answers. This result does not match the expectation derived from a music analysis made only on the score. It is also interesting to note that the two accents with the higher frequency of answers are characterized by the change (Ch): a tonal change in the first case, and change of register in the second case. In addition, the HF accents divide the piece into three different parts, and each of them is characterized by one of the three types of RF. In conclusion, the HF accents indicate the perceptual macroform of the piece (OS).

## Computational Modeling

### Method

The purpose of our computational model was to predict listeners’ responses (dependent variables) from a set of underlying principles (i.e., features used as independent variables) extracted from score information. The modeling procedure consisted of four steps: (1) The data from the listening test were averaged in order to provide a single value for each note, corresponding to the perceived accent. (2) We defined a large set of basic local features related to the metrical, rhythmical and melodic structure, starting from previous studies (e.g., Friberg et al., 1998; Müllensiefen et al., 2009; Bisesi et al., 2019), and the manual music analysis presented in section “Music Analysis.” (3) We formulated a prediction method by using linear or support vector regression. (4) By isolating specific groups of features and single musical styles, their respective independent contribution was estimated in relation to the overall prediction results.

### Features

Five groups of features were used. *Pitch Contour* features relate to the pitches of the melody disregarding any rhythmic information, *Tempo* features relate to note IOIs (interonset intervals), *Timing* features relate to the local variation of note durations (IOIs), *Simple Phrasing* features relate to melodic groups or fragments and finally *Meter* features relate to the different metrical levels as defined by the score. A list of all features together with a short description is provided in Table 4.

**Table 4:** List of the final features and a short description.

Number	Feature name	Description
		
	*Pitch Contour*	
1	f0_pos_dist_mean	Positive distance to running pitch mean (semitones)
2	f0_neg_dist_mean	Negative distance to running pitch mean (semitones)
3,4	f0_bef_pos_leap_p (_log)	First note of positive leap (1/0 or log leap size)
5,6	f0_bef_neg_leap_p (_log)	First note of negative leap (1/0 or log leap size)
7,8	f0_aft_pos_leap_p (_log)	Second note of positive leap (1/0 or log leap size)
9,10	f0_aft_neg_leap_p (_log)	Second note of positive leap (1/0 or log leap size)
11	f0_aft_leap2_log	Third note in a four-note context with leap in the middle and with an up-down-up or down-up-down pattern (log leap size)
12	f0_bef_leap2_log	The same as above but marked on second note (log leap size)
13,14	f0_pos_peak_p (_log)	Positive peak in three notes, one before one after (1/0 or log leap size)
15,16	f0_neg_peak_p (_log)	Negative peak in three notes, one before one after (1/0 or log leap size)
17	f0_pos_peak2_p	Positive peak in four notes, two before one after (1/0)
18	f0_pos_peak3_p	Positive peak in five notes, three before one after (1/0)
19	f0_first_arp_up_p	First note in upward arpeggio, two up-leaps preceded by any other int. (1/0)
20	f0_last_arp_up_p	Last note in upward arpeggio (1/0)
21	f0_first_arp_down_p	First note in downward arpeggio (1/0)
22	f0_last_arp_down_p	Last note in downward arpeggio (1/0)
		
	*Tempo*	
23	dr_ndr	IOI (ms)
24	dr_very_short_note	Very short notes (0–1)
		
	*Timing*	
25,26,27	dr_short_before_p (rel,_log)	On long note after short (1/0, rel, log)
28	dr_short2_before_p	On long note after two equally short notes (1/0)
29	dr_short3_before_p	On long note after three equally short notes (1/0)
30	dr_short_after_p	On long note before short (1/0)
31	dr_first_short_p	First of at least two short notes (from punctuation) (1/0)
32,33	dr_longest_five_p (_w)	Longest in the middle of five notes (1/0 or gradual weight)
34	dr_short_between_long_p	A short note between longer (1/0)
35	dr_long_after_p	Long note after (inhibit feature) (1/0)
		
	*Simple phrasing*	
36	ph_rest_before_or_first	Note after rest or the first note
37	ph_rest_after_or_last	Note before rest or the last note
38	ph_punct_first	First note of small melodic fragment (from punctuation) (1/0)
39	ph_punct_last	The same for last note (1/0)
		
	*Meter*	
40	beat0	Sub-beat (1/0)
41	beat1	Beat or tactus level (1/0)
42	beat2	Half bar or bar (1/0)
43	beat3	Bar or 2 bars (1/0)

*The resulting values are indicated in parentheses. The extension “_p” means a logical variable and any other extension indicates a varying weight value that is either logarithmic (_*log*), relative (_*rel*) or weighted (_*w*)*.

Most of the previous studies mentioned in the introduction (e.g., Friberg et al., 1998; Müllensiefen et al., 2009; Bisesi et al., 2019) have features related to, in particular, melodic leaps, peaks, and rhythm using different formulations. Instead of following a strict formulation as in the previous studies, we left our formulation relatively open and suitable for extending all aspects, thus our aim was to define a set of features that would be less prone to a specific assumption. In this way, our system has been allowed to select and optimize the use of features autonomously by means of machine learning and according to the responses of the listeners. The same approach was followed for implementing the suggestions from the music analysis presented in the Section “Music Analysis.”

One possible influence for many features is provided by interval size or note duration. For example, it is likely that the influence of a pitch leap will depend on the leap size in relation to the surrounding intervals. In order to accommodate this in a simple fashion, several features were defined in two or three versions, one with a binary response (no influence of intervals or durations), and one with a weight according to the size of the interval or the duration. These versions are marked with the different extensions in parenthesis in the table. In the final analysis, only one of each was selected.

Most of these features were defined from the local context of a few notes that might be considered a relatively low level of processing. The examples of a relatively higher-level perception are the simple phrasing and the meter features. Several other musical aspects that could be hypothesized to contribute to the perceived accent, such as the harmonic structure, have not been included in the current formulation. Such musical concepts are less straightforward to be included since they depend on perceptual data and/or music-theoretic analysis.

In Friberg et al. (1998), a model for melodic punctuation is described. That model used a set of 13 sub-rules for determining the boundaries of small melodic units and mark them in the performance by inserting a micropause after the last note of each unit. Several of these sub-rules were used in the current Pitch Contour and Timing feature groups. In addition, the identified boundaries were also used as features in the Simple Phrasing group.

#### Pitch Contour

We chose to divide the features into a positive (ascending interval) and a corresponding negative (descending interval) feature for each principle, although Thomassen (1982), for instance, found relatively small effects as a function of the interval direction.

Features 1 and 2 emphasize any note that is high or low in comparison with the running mean pitch of the melody computed for the last two measures or at least 10 previous notes (the same as in Bisesi et al., 2019). This is a gradual measure with the resulting weight in semitones from the mean.

Features 3–12 all consider the first or the second note in a pitch leap (interval >2 semitones). Features 3–10 only consider the leap itself with the different features for the first or second note as well as positive or negative leaps. Thus, the context is the smallest possible—consisting only of two notes. The gradual weight for all four cases is computed as log_2_(|ΔST|-1), where ΔST is the interval size in semitones. The logarithm here is used in order to diminish the influence of very large intervals.

Features 11 and 12 are a simplification of a previous pitch context rule for finding the boundaries of small melodic groups (“Subrule 3. Leap” in Friberg et al., 1998; see also “jumploc” in Müllensiefen et al., 2009). Thus, it is assumed that a perceived accent can occur on the first or last note of a leap. The context is four notes and thus three intervals. The middle, target interval has to be a leap and larger than the other intervals. The direction must be either down-up-down or up-down-up. The weight is equal to log_2_(|ΔST|-1) as above. The two features 11 and 12 apply the same weight on either the first or the second note of the middle interval.

Features 13–18 consider local peaks or troughs in the pitch profile. Features 13–16 use a three-note context of which the middle one needs to be the highest or lowest, respectively. The versions with gradual weights are computed as log_2_(|ΔST| +1), where ΔST is the first interval. Features 17 and 18 use a somewhat larger context of either four or five notes. Only positive peaks and logical weights are defined.

Features 19–22 consider the first or last notes of an arpeggio of any length using a four-note context. For example, for the detection of the first note of the upward arpeggio, the target is the second note of the four-note context, the first interval could be anything except an upward leap, and the two following intervals should be both upward leaps.

#### Tempo

Features 23 and 24 are both related to tempo in an indirect way. Feature 23 (dr_ndr) is just the IOI of each note. The assumption is that relatively more notes are accented when the tempo (or rather the number of notes per second) is low. Feature 24 (dr_very_short_note) considers very short notes that are either shorter or on the boundary of being perceived as individual events (Friberg and Sundström, 2002; London, 2012; Polak, 2017). The weight = 1 for 0 < IOI < 50 ms, a linear slope from 1 to 0 for 50 < IOI < 150 ms, 0 for IOI > 150 ms.

#### Timing

Features 25–35 (dr_...) are all related to differences in IOIs between adjacent notes in a small context of a few notes. Most of them reflect in various ways the basic idea that a relatively long note within the context of relatively shorter notes is accented. Feature 35 identifies a short note followed by a long one; thus, it could potentially enhance the accent on a long note by inhibiting the accent on the preceding short note. It can also identify patterns in which the accent appears on the short note.

Features 25–27 (dr_short_before…) trigger on a longer note after a relatively shorter note. Three different versions are defined: binary, the relative difference between IOI_short_ and IOI_long_ with weight = 1- (IOI_short_ / IOI_long_), and a logarithmic version with weight = ln (IOI_short_ / IOI_long_).

Features 28 and 29 just extend the context of 2–3 preceding shorter notes. Feature 30 is the corresponding version when the long note is followed by a shorter one.

Feature 31 (dr_first_short_p) gives a binary weight to the first of a series of short notes that follows after a longer note (Friberg et al., 1998).

Features 32 and 33 (dr_longest_five…) mark the longest (middle) note in the context of five notes. The gradual weight is computed according to the specification in Friberg et al. (1998).

Feature 34 (dr_short_between_long_p) adds a binary weight to a short note surrounded by longer ones, a rule in the KTH performance system (rule 6 in Sundberg et al., 1983; see also e.g., Friberg et al., 2006).

Feature 35 (dr_long_after_p) is chosen so it can work as an inhibition feature preventing an accent from being marked on a note preceding a longer note.

#### Simple Phrasing

Features 36 and 37 (ph_rest...) mark each note before or after a rest. Thus, they can be viewed as a kind of a simple low-level phrasing indicator. Features 38 and 39 correspond to the first and the last note of the small melodic units identified by the punctuation rule (Friberg et al., 1998).

#### Meter

Features 40–43 mark four different metrical levels. The meter analysis was based on the notated meter in the original score. The different metrical levels (beat0-beat3) are computed according to Supplementary Table S2. These features mark each note that appears on one of these metrical levels with the corresponding label. The list in Supplementary Table S2 covers most of the basic meters, as well as the more unusual ones found in the database. The marking of the metrical levels is an extended version of Bisesi et al. (2019). However, the specific salience values are not computed and instead left to the machine learning model for optimization. Since the presented melodies lacked the original accompaniment (as specified in the original score), the perceived meter might not correspond to this notated meter for all melodies. However, the perceived meter can only be obtained using the same examples in a separately conducted experiment. Nevertheless, we assumed that in many cases this was a reasonable approximation.

**Figure 5:**
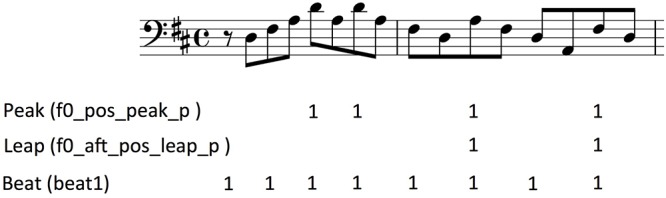
An example of how three binary features are applied to the first two measures of melody no. 1, Toccata for solo cello by Vitali. These binary features mark the note that is triggered by the context with the value 1; all other notes are marked with 0 (not shown).

An example illustrating the output of three binary features is shown in Figure 5. All the features with a gradual output weight in Table 4, inserts instead the corresponding weight value on the target note.

### Comparison Between Musical and Computational Features

Let us compare the list of musical features found in the music analysis Section “Preliminary List of Musical Features of Perceived Accents” with the features defined in Table 4.

Repeated Fragments (RF), describing short melodic fragments which are repeated along the piece, is a potentially important principle but is difficult to implement as an automatic feature as it requires an automatic fragment/grouping analysis. There exist several such systems (e.g., Friberg et al., 1998; Ahlbäck, 2004), but they also introduce analysis errors. Here, we chose both to include some of the underlying features used in Friberg et al. (1998) for determining small melodic groups (Features 11, 12, 31–33 in Table 4), as well as the final identified melodic fragments (Features 38 and 39). However, detection of fragment repetition was not explicitly implemented.

Arpeggio notes (AR), which describe groups of ascending or descending notes in arpeggios, are partially implemented by the arpeggio features (Features 19–22). In this case, they only consider the first or last note of an arpeggio (i.e., not all notes).

Notes on main beats (BE) and strong beats (SB) correspond to the Meter features (Features 40–43 in Table 4).

The accents on the weak beats (WB) were not explicitly implemented, but could potentially be detected by the model as a combination of meter and, for example, comparatively long notes (Features 23, 25–30, 32–33).

Long (LG) has several corresponding features in the Timing feature group (Features 25–30 and 32–33).

Climax (CL), which refers to notes concluding an ascending or descending fragment, that is the highest or the lowest pitch of the fragment, partially corresponds to the melodic peak features in the Melodic Contour group (Features 1 and 2, 13–18, 20, and 22).

Last and First note of a melodic fragment (LN, FN) partially correspond to the simple phrasing features (Features 36–39) with both the features marking notes before or after a rest (Features 36 and 37), and the features detecting small melodic fragments taken from Friberg et al. (1998) (Features 38 and 39).

Notes introducing a strong variation (Ch) have a coupling to the leap features in relation to register and to the rhythmic features; however, changes in for example tonality were not considered.

Embellishment (EM) was not implemented directly but should have a coupling to the Tempo features (Features 23 and 24).

Features concerning the overall structure (OS) were not explicitly included since there was no direct mechanism for detecting the phrase structure at this level. However, due to the abundance of features signaling an accent at these positions (at least in the example of Figure 4), it should be possible for the system to identify these positions.

For tension and relaxation (T/R), an automatic implementation was not possible since this would require a more detailed and complex automatic music analysis of melodies.

Syncopation (S) and anacrusis (A) were not explicitly included but might be detected as a combination of other features, such as the Meter and Timing features.

Finally, HAR features (important harmonic functions like modulation, dissonance, etc.), C (end of a cadence), and T (tonal functions: tonic, dominant, etc.) were not included in the model since manual or automatic analysis of the harmonic structure were not included in this study.

Hence, a majority of the features identified in the music analysis, although not all of them, have been implicitly or explicitly included in the computational model developed in this study. The main exception consists of the features related to harmonic function and to tension/relaxation.

### Prediction Methods and Training

In the selection of the prediction methods, we chose to use both a simple and a more advanced model. Due to the relatively small number of features (34 after selection) in relation to the total number of cases (4204), Multiple Linear Regression (MLR) could be used as the first method. MLR has the advantage that it is easy to estimate the contribution from each feature. However, obviously, due to its definition, this method does not account for any interaction among features, something that was hypothesized as being important. Therefore, as a second method we applied Support Vector Machine regression (SVR) using the LIBSVM version 3.22 package for Matlab (Chang and Lin, 2011). A radial basis function was used as the kernel, and the model parameters C and gamma were optimized using a grid search. All models were evaluated using 10-fold cross-validation, taking the average result over 10 random repetitions.

## Results

### Correlation Analysis and Feature Selection

For the features that were defined with several different output weights, a selection was made by computing the correlation between all features and the overall mean ratings for the test set. For all correlations we used either the point-biserial or the Pearson correlation depending on whether the feature weight was binary or gradual. For each of these features, the version with the highest correlation was retained in the subsequent feature set. This resulted in a total of 34 features (see Table 5).

**Table 5:** Results of the correlation and MLR analyses.

	Correlations with mean ratings		Multiple linear regression		
	
feature	all participants	optimized group	beta	*sr*	*p*-value
*Pitch Contour*					
f0_pos_dist_mean	0.17***	0.15***	0.150	**0.120**	0.000***
f0_neg_dist_mean	0.12***	0.10***	0.147	**0.117**	0.000***
f0_bef_pos_leap_p	-0.05**	-0.03	0.006	0.004	0.658
f0_bef_neg_leap_p	-0.05**	-0.01	-0.040	0.031	0.002**
f0_aft_pos_leap_log	0.10***	0.12***	0.027	0.016	0.095
f0_aft_neg_leap_log	0.08***	0.09***	0.047	0.029	0.003**
f0_aft_leap2_log	0.14***	0.12***	0.034	0.027	0.005**
f0_bef_leap2_log	0.04**	0.05***	0.016	0.014	0.161
f0_pos_peak_log	0.13***	0.13***	0.085	0.043	0.000***
f0_neg_peak_log	0.07***	0.05**	0.091	0.054	0.000***
f0_pos_peak2_p	0.16***	0.16***	-0.006	0.003	0.767
f0_pos_peak3_p	0.16***	0.17***	0.102	0.052	0.000***
f0_first_arp_up_p	-0.04**	-0.01	-0.019	0.016	0.095
f0_last_arp_up_p	0.06***	0.08***	-0.004	0.004	0.693
f0_first_arp_down_p	-0.01	-0.01	0.008	0.007	0.446
f0_last_arp_down_p	-0.01	0.02	-0.052	0.046	0.000***
*Tempo*					
dr_ndr	**0.43**^∗∗∗^	**0.55**^∗∗∗^	0.212	**0.165**	0.000***
dr_very_short_note	-0.07***	-0.19***	-0.042	0.038	0.000***
*Timing*					
dr_short_before_p	**0.48**^∗∗∗^	**0.57**^∗∗∗^	0.219	**0.125**	0.000***
dr_short2_before_p	0.39***	**0.46**^∗∗∗^	0.064	0.036	0.000***
dr_short3_before_p	0.33***	0.37***	0.018	0.013	0.199
dr_short_after_p	0.31***	**0.42**^∗∗∗^	0.094	0.050	0.000***
dr_first_short_p	-0.09***	-0.14***	-0.041	0.033	0.001***
dr_longest_five_w	0.32***	0.38***	0.038	0.025	0.012*
dr_short_between_long_p	0.17***	0.22***	-0.002	0.002	0.862
dr_long_after_p	-0.06***	-0.17***	-0.004	0.004	0.693
*Simple Phrasing*					
ph_rest_before_or_first	-0.01	0.05**	0.106	0.083	0.000***
ph_rest_after_or_last	0.26***	0.31***	0.187	**0.121**	0.000***
ph_punct_first	-0.06***	-0.08***	-0.019	0.014	0.164
ph_punct_last	0.39***	**0.51**^∗∗∗^	0.046	0.024	0.013*
*Meter*					
beat0	0.18***	0.28***	0.026	0.020	0.039*
beat1	0.30***	**0.40**^∗∗∗^	0.108	0.073	0.000***
beat2	0.28***	0.36***	0.107	0.066	0.000***
beat3	0.19***	0.26***	-0.007	0.005	0.591

*Correlations between final features and mean ratings for all participants and for the optimized group (columns 2 and 3). *r* ≥ 0.4 emphasized in bold. Detailed results from the MLR model predicting the optimized group with beta-weights, semipartial correlation coefficient *sr* and *p*-values (columns 4–6). *sr*-values > 0.1 are marked in bold. Significance levels ^∗^*p* < 0.05, ^∗∗^*p* < 0.01, ^∗∗∗^*p* < 0.001*.

Note that the “log” versions were selected for the contour features targeting a weight on the second note in leaps and peaks. The binary versions were selected for the first note in a leap. However, note that the correlations for these features were negative, indicating a suppression of an accent on these positions. For the timing feature with a rather simple context (dr_short_before_p), the binary version was selected over two different gradual versions. This indicates that the IOI relation between the short and the long note is less important—just a difference is enough. For the more complex timing feature dr_longest_five_w, the version with the weight according to the previous study was selected.

The correlations listed in Table 5 (column 2 and 3) have been computed for the whole data set. Due to the multiple testing, the significance levels should be interpreted with some caution and should be viewed only as an overall indication of the correspondence. As seen in the table, with a few exceptions, most features are correlated to some extent with the ratings. This makes it problematic to exclude any feature at this stage on the basis of the correlations. The highest correlations are marked in bold. There are also a few features with rather weak coupling to the ratings. As mentioned above, the first note in a leap seems not to be supported; additionally, the first short note in a series (dr_first_short_p) and first note after rest (ph_rest_before_or_first) have negative or weak support. For the arpeggio features, only the last note in upward arpeggio is significant (f0_last_arp_up_p). Relatively high correlations are found for some surprisingly simple features, like note IOI (dr_ndr) and long after short (dr_short_before_p). A relatively high correlation is also obtained for the last note in short melodic fragments (ph_punct_last). For the meter features, the highest *r* is obtained for the metrical beat/tactus level (beat1). All metrical levels get modest, medium *r*-values indicating that there is a significant contribution from the meter. This may also indicate that the notated meters are a reasonably close approximation to the perceived meter. Notice also that the correlations are in general higher for the optimized group.

The same correlations were also computed for each feature and each individual rating by the 30 participants (not shown in the table). The overall correlation across all features, as estimated by the mean |*r*|, varies greatly across the participants and ranges from 0.034 to 0.178, indicating again that either the strategies and/or the consistency varies across raters. We will investigate this further using the model below.

### Overall Results

Both MLR and SVR were applied using the reduced feature set both for all raters and for the optimized group. We used 10-fold cross-validation with 10 random repetitions using the whole dataset. The overall results in terms of the explained variation *R*^2^ are shown in Table 6. *R*^2^ is defined as the squared correlation between the mean ratings and the prediction of the model across all notes. We see that we get rather modest overall results, reaching 66% for the best case. The SVR method performs better than MLR, as expected. Notice that there is a relatively large improvement for the optimized group in comparison to all raters. An example of the model results using the SVR method without cross-validation (thus trained on the whole dataset) is shown in Figure 2 for music example no. 1. As seen in the figure, the model retains many of the main characteristics of the accent pattern, in particular regarding the relatively stronger accents, while the details between these vary more.

**Table 6:** Overall modeling results using Multiple Linear Regression (MLR) and Support Vector Regression (SVR) in terms of explained variance *R*^2^.

	*R*^2^ all raters (%)	*R*^2^ optimized group (%)
MLR	43.3	59.1
SVR	52.8	66.0

The MLR method was also applied without any cross-validation. The corresponding *R*^2^-values were 44.5 and 60.0% for all raters and the optimized group, respectively (not shown in Table 4). This small difference of about 1% for all raters indicates that there is no problem with over-fitting of the MLR model; thus, all the features can be retained as they are and the model without cross-validation can be used for a feature analysis as presented in the next section.

### Feature Analysis Using MLR

In order to investigate the influence of each feature, the detailed results of the MLR applied on the whole dataset without cross-validation are shown in Table 5. Since the *R*^2^ for all raters were quite low, we show only the results for the optimized group. All features are listed along with the beta-weights, the semipartial correlation coefficient *sr*, and the corresponding *p*-values. The *sr* coefficient reflects the independent contribution of each feature. The highest contributions in terms of *sr*-value are shown in bold. It is not surprising that none of the pitch contour features gets a high *sr*-value since the system may combine the features in different ways if one feature is missing, thus “covering up” for the missing feature. This is natural, given the presumably high correlation between some features.

In comparison with the correlations, there are several interesting observations. The features for the first note of a leap (e.g., f0_bef_pos_leap_p) obtained a small *sr*-value and also a small correlation, and thus, seem to be of less importance. The corresponding feature for the second note in a leap (e.g., f0_aft_pos_leap_log) obtained a rather low *sr*-value compared to the correlation. Apparently, the different leap rules in the MLR analysis are combined in different ways, changing the significance and impact in comparison with the correlation analysis. There is a similar effect for the Timing features. There are 3–5 features with a relatively high correlation (dr_short_before_p, dr_short2_before_p, dr_short3_before_p, dr_longest_five_w dr_short_after_p). In the MLR analysis, only the first one remains with a relatively higher contribution. Thus, the rather high correlations among similar features are to a certain extent resolved in the MLR analysis.

### Influence of Feature Groups and Styles

The influence of different feature groups and styles was investigated in more detail. In the overall results, it was found that the best results were obtained for the SVR model and the optimized rater group. Thus, we used only this combination here since it could be expected to give the results a relatively higher validity. The features were the reduced set consisting of 34 features in total, divided into the five groups. All computations were performed using 10-fold cross-validation with 10 random repetitions.

The overall resulting explained variance in terms of *R*^2^ for the different musical styles are shown in the second column in Table 7. Rather small variations are obtained compared to the 66.0% obtained for the whole set. There is a slightly higher *R*^2^ for Post-tonal style, slightly lower *R*^2^ for the Baroque style, and a slightly lower *R*^2^ for Vocal versus Instrumental.

**Table 7:** SVR modeling results in terms of *R*^2^ for different styles and feature groups.

	Model *R*^2^ (%)	Independent contribution of each feature group (%)			
	
Music selection	All features	Pitch contour	Tempo	Timing	Simple phrasing	Meter
All	66.0	9.6	4.5	7.0	4.4	3.9
Baroque	62.2	6.0	4.6	4.8	3.6	5.4
Romantic	64.8	8.7	2.7	8.5	3.4	3.1
Post-tonal	69.1	11.3	5.1	7.1	3.4	2.2
Instrumental	67.3	11.5	3.1	4.7	4.8	4.6
Vocal	62.0	7.2	4.8	5.2	2.3	2.8

To investigate the effect of different feature groups, one group was removed and the model was optimized for the remaining features. The difference in *R*^2^ between the full model and the reduced model was used as a measure to estimate the independent contribution from each feature group. This is similar to the squared *sr*-value in MLR, although those numbers cannot be directly compared. The result is shown in Table 7, columns 3–7, for all music examples and styles. Note that the percentages do not add up to the result for all features since they only estimate the independent contribution.

In general, we can observe that Pitch Contour features provide the highest contribution (9.6% overall) followed by Timing features (7.0% overall). Tempo, Simple Phrasing, and Meter features have a comparatively smaller contribution of around 4% (range 3.9–4.5). The higher contribution for Pitch Contour and Timing is not surprising given that these groups contain more features and are also central in most previous studies. More surprising is the contribution of Tempo. Possibly, from the results of the correlation and MLR, there is only one contribution feature, i.e., the IOI of the note without considering any context (dr_ndr). Thus, in general, longer notes are more accented even if the context within each melody is disregarded. The overall contribution by Meter is surprisingly low considering the importance of meter for melody perception according to previous research.

The influence of the feature groups for different styles has a few interesting variations. We can see that the influence of Tempo is relatively small for the Romantic style (2.7%) and relatively large for the Post-tonal style (5.1%). The influence of Timing is relatively small for the Baroque style (4.8%) and relatively large for the Romantic style (8.5%). The influence of Meter is relatively small for the Romantic style (3.1%) and also for the Post-tonal style (2.2%). The latter is not surprising given that for many examples in the Post-tonal group, a regular meter is not suggested. There are several differences between the Instrumental and Vocal groups. This is perhaps not surprising given that vocal melodies are often slower with longer notes. One example is the difference in Pitch Contour, which contributes 11.5% for the Instrumental group but only 7.2% for the Vocal group.

### Individual Modeling of Each Participant

The SVR model was also used to predict the ratings of each participant separately; see Table 8. The method was the same as before, using the reduced feature set with 10-fold cross-validation and 10 random repetitions. The contribution from each of the five feature groups was also estimated as above. Here we only made the calculations for the ten raters with overall *R*^2^ > 20 %, to ensure some validity of the results.

**Table 8:** SVR modeling results for each individual participant in terms of *R*^2^.

			Model *R*^2^ (%)	Independent contribution of each feature group (%)		
**Rater**	**Musical experience**	**Optimized group**	**All features**	**Pitch contour**	**Tempo**	**Timing**	**Simple phrasing**	**Meter**

1	4.1		8.8					
2	2.1		1.5					
3	2.0	X	**41.8**	11.6	1.8	8.7	1.7	8.8
4	2.9		8.9					
5	3.7	X	12.1					
6	3.4	X	**35.8**	2.5	7.4	2.7	3.3	0.3
7	2.4	X	7.8					
8	2.1		10.1					
9	2.5		0.3					
10	2.3	X	**30.8**	4.5	2.1	5.3	4.5	3.4
11	2.3	X	**24.9**	7.6	2.6	7.3	1.6	3.1
12	3.1	X	**39.3**	15.2	2.9	1.2	5.4	3.9
13	2.9	X	15.4					
14	3.6		4.2					
15	2.4		-6.0					
16	2.0		**20.5**	5.9	10.9	5.8	1.1	2.1
17	1.7		0.6					
18	3.1		-10.2					
19	1.7		-1.7					
20	1.9		10.2					
21	2.3	X	6.0					
22	4.4	X	**20.5**	7.4	2.2	5.2	1.6	2.2
23	4.4	X	**38.9**	9.3	1.3	2.8	1.7	6.4
24	3.4		-2.6					
25	2.5	X	**25.2**	10.1	4.0	5.3	4.3	2.6
26	3.3	X	12.4					
27	5.0		12.7					
28	4.4	X	**40.9**	9.1	1.6	4.9	1.3	5.7
29	2.2	X	16.2					
30	4.3		10.2					

*The participants in the optimized group are marked with an X. The influence of feature groups is presented only for the resulting *R*^2^ > 20% (marked in bold)*.

Interestingly, the overall *R*^2^ varies greatly among participants, with the highest *R*^2^ = 41.8 (Rater 3) and the lowest below zero. Negative values are possible since the model uses cross-validation. This indicates that the model could not find any systematic generalization using the suggested features for these participants.

Looking at the contribution of feature groups, we can clearly observe a difference in the strategies among participants. For example, if we compare Raters 3 and 6, Rater 3 has a strong coupling to Pitch contour, Timing and Meter features, while Rater 6 shows an almost opposite pattern with stronger emphasis on Tempo and Simple Phrasing. Note that these percentages are not directly comparable between participants since they also depend on the overall *R*^2^.

There was a significant correlation between optimized group (coded as 0/1) and Model *R*^2^ (*r*_pb_ = 0.70, *p* < 0.001). There was no significant correlation between either musical experience and model *R*^2^ (*r* = 0.20) or between musical experience and optimized group (*r* = 0.1).

## Discussion

Several topics emerged from the results of this study. They mainly concern its original methodology, the listening experiment, the music corpus, the music analysis, the computational model, and finally the relation between the music analysis and the model.

In the listening experiment, where the participants marked the perceived accents, we collected a large set of empirical data about the perception of immanent accents. This database organizes the empirical data on the basis of vocal and instrumental music, and in three different historical styles. The overall agreement in terms of Cronbach’s alpha (CA) was 0.836 and the mean pairwise correlation was 0.157. Given this relatively large variability among the raters, we made an automatic optimization of CA in which participants who lowered the final value were omitted. This resulted in an optimized group of 15 raters with *CA* = 0.861 and the mean pairwise correlation = 0.303.

The use of a selected musical repertoire with the control of the two variables style and genre (Vocal and Instrumental) allowed us to investigate the perception of immanent accents in differentiated but controlled musical contexts. The music analysis of the answers by selected individual raters showed that different styles produced differences in the kind of accent that was prevalent. For example, the repetitive and chordal Baroque structure produced dominantly repeated fragments (RF) and arpeggio notes (AR). The French melodies gave prominence to embellishments (EMs). In the Romantic style, the perceived accents seemed to depend on more complex, personal and irregular structures, and more “uncertain” categorizations of accents (UA) were also present. In the Post-tonal style, a difference was observed between melodies composed in the first 50 years of the 20th century and melodies written in the second half of the century. Other accent indications were more equally distributed among the different melodies. Instead, small differences were found for different musical styles by means of the computational model. However, the impact of the styles and genres on the perception of immanent accents still remains to be fully analyzed by means of an analysis of all 60 melodies used in the experiment.

The music analysis of rated accents indicated a number of features related to interconnected notes (repeated fragments, arpeggio notes, notes on main beat), single notes (strong and weak beats, long notes, phrasing, cadence, climax, embellishments), and overall structure (overall structure, tension and relaxation). In addition, there were a number of accents to which no plausible explanation could be found (uncertain accents). Some variability among the raters was found that was difficult to explain more than to ascribe it to individual preferences. It was also evident that different styles and different composers put different emphasis on different principles depending on the musical structure. The analysis of the “strong” accents by all participants carried out with a selected number of melodies allowed us to observe some common features, which characterize the accents indicated by the majority of the participants. We can argue that these common features could be more predictive than those used only by single raters. There were one or two accented notes with a higher frequency of answers (HF) of answers in each song. They were characterized by changes in register, strong beat, climax, and corresponded to the first or last note of a melodic fragment. Most important, in all the melodies analyzed, the HF notes divide the piece into parts characterized by the presence of one or two kinds of RF. Thus, HF accents work as cues that affect the perception of the overall structure of the piece. These results are in line with our previous studies on the memorization of the overall structure (Addessi and Caterina, 2000, 2005). Instead, surprisingly, it seems that the strong beat (SB) does not affect the perception of the strong accents more than the weak beat (WB). In fact, in the three examples presented above, the SBs are 18.8% of the notes, while the WBs are 19.7%. The accents on long notes (LG) appear in a metrically strong and tonally important position, almost always in a cadence position. The climax notes (CL) are often accented but not always. Many notes are accented at the beginning and end of a melodic phrase: the phrasing, therefore, seems to affect the perception of immanent accents. The changes (Ch) are important for the perception of accents. The embellishments (EM), where they are present, always induce the perception of accents, but they have mostly low frequency (LF) of answers. These results suggest that a formulation of features for the computational model should take into account both local musical principles and the overall structure of the pieces, the tension and relaxation dynamic (see for example Bigand, 2003), as well as the principles derived from the cognitive processes of perception of similarities/differences and repetition/variation/contrast (see for example Deliège, 2007). A further music analysis of the marked accents of all 60 melodies would presumably give more data and insights to better describe this list of musical features of perceptual accents.

In the modeling, the best results were obtained using SVR, with an explained variance of 66.0% for the optimized group using 10-fold cross-validation. Using this method, the influence of five different feature groups was found to be about 9.6 and 7.0% for Pitch Contour and Timing, 4.5% for Tempo, 4.4% for Simple Phrasing, and 3.9% for Meter. Small differences were found for different musical styles. For example, the model could predict the accents to a higher degree for Post-tonal music compared to the other styles and the Instrumental group was better predicted than the Vocal group (Table 7). For the interaction between feature groups and styles it could be observed that the accents in the Post-tonal style, in comparison with the other styles, received a relatively smaller influence from Meter and a higher influence from Pitch contour and Tempo. Also, for the Romantic style, the Timing group was more prominent than in the other two styles.

The modeling of the individual raters revealed large differences in the adopted strategies. For example, Rater 3 relied strongly on the Meter, Pitch Contour and Timing, while Rater 6 used the almost opposite pattern with no influence from Meter. This clearly shows that listeners focus on different features and, thus, perceive the same melody in different ways (c.f. Tervaniemi et al., 2016). Also remarkable was that several raters could not be modeled at all using the suggested features, implying that they used completely different features or a non-predictable response in relation to the music structure.

The musical structure of each melody in the current sample is evidently important since the musical context of each feature needs to be found in the piece for the feature to have any effect. For example, some melodies or passages contain only one note value (e.g., first part of Vitali, melody no. 1). Obviously, none of the timing features will be triggered in this case. The comparison among the different styles is therefore dependent on the selection of the examples in each stylistic group. Presumably, using a larger corpus of melodies would give more representative results for each style.

The highest overall modeling result was *R*^2^ = 0.66, predicting the mean of the optimized group. Due to the many differences in methods, comparison with other studies is limited. The most similar study is Müllensiefen et al. (2009), who used another definition of accuracy but computed also the Pearson correlation, which in the best case reached *r* = 0.7, implying a corresponding *R*^2^ = 0.49 when using the ratings of all participants. In fact, despite many differences in the methods, in this study we obtained a value of *R*^2^ = 0.53 using the mean of all participants. The results in Bisesi et al. (2019) indicate a similar level of agreement with *r* = 0.66 corresponding to *R*^2^ = 0.44 for the best models. Note, however, that Bisesi et al. used a totally different approach based on a top-down formulation that includes and combines three main accent categories—metric, melodic and harmonic—instead of being a bottom-up rule detection by means of machine learning techniques. Nevertheless, these results indicate that it might be difficult to reach a high accuracy with any model if many participants are combined (for example by mean values), since the variation among participants is rather large. Instead, if we make a selection of the participants, as in the optimized group in this study, a substantial improvement can be obtained.

As shown in Figure 2 for one music example, the overall qualitative shape of the model prediction is similar to the mean rating across participants. In this example, all the relatively stronger accents are in fact correctly identified, while most of the variation is related to the relatively weak (less important) accents. Note that even single misplacements (an accent on the wrong note) of the predicted accents will lower the overall accuracy substantially. As an example, consider a melody consisting of 70 notes with 15 notes having a binary accent (1/0). If the model predicts these accents correctly but with one of them on the wrong note, the *R*^2^ goes down from 1 to 0.86, thus a reduction of 16% in accuracy when one out of 70 notes is wrong (corresponding to 1.4% of the notes or 6.7% of the accented notes).

In the present study, we have used meter and phrasing as input to the accent modeling. However, it is likely that the perceptually formed accents serve rather as the *starting point* for the perceptual formation of higher-level concepts, such as phrase macrostructure, meter, and key (Müllensiefen et al., 2009; Koelsch, 2011). Which one came first from a perceptual point of view is difficult to verify in behavioral experiments since we only get the response after all mental processing. Presumably, the process is both top-down and bottom-up, making a more accurate modeling of, in particular, individual responses a rather complex task. Support for a dominant bottom-up processing is provided by the relatively low contribution from the meter group of features. However, as discussed previously, our annotations of meter are taken from the score and not from the perceived meter in these melodies (presented without accompaniment). This potential mismatch could also contribute to a lower contribution from the Meter group.

A part of the variation could be explained by neither the music analysis nor by the model. It could be that some of the participants used different strategies that were not identified either in the manual or automatic analyses. Presumably, the reason could also be that they interpreted the task in a different way, that they changed strategy over time, or just that the notes did not “pop up” as different to each other, making individual responses become more random. An extended music analysis of all 60 melodies may reveal additional strategies used by the participants.

Concerning the relation between music analysis and computational model, it was possible to implement several features emerging from the music analysis into the computational model. These features increased the predictability of the model. However, several features were not yet implemented, concerning both harmonic function and tension/relaxation, and some detailed features on phrasing and rhythmical structures. Also, many of the implemented features were only indirectly or implicitly connected to the music analysis features. An improved description of the features for perceived immanent accents represents a major challenge both in the modeling process and in the music analysis. One of the reasons is that the perception of accents relies on the psychological processes of listening in real time. Also, a precise formulation of a feature able to automatically identify the musical context is difficult. We hypothesize that the combination of an improved implementation of existing features and the inclusion of additional features that might emerge from the analysis of all melodies will contribute to enhance the predictability of a future computational model.

## Conclusion

In this study, we addressed the question of how the perception of “immanent accents” (Parncutt, 2003) can be predicted and modeled. Using a “mixed methods” approach that combines computational modeling, musicology, music analysis, and experimental methods, as well as suggestions from previous studies (e.g., Friberg et al., 1998; Müllensiefen et al., 2009; Bisesi et al., 2019), we were able to identify a large number of underlying musical features and use them to model the perceived immanent accents.

The listening experiment produced a large and unique collection of perceptual and empirical data about the perceived immanent accents, organized in three different musical styles belonging to the Western art music repertoire, as well as in vocal and instrumental genres. The complete data set contains the answers of 30 participants, who marked the perceived accents on each of the 4204 notes, totaling 126,120 data points.

The music analysis and the method that we used, which was based on the analysis of the musical features characterizing the accents indicated by the listeners, contributed to implement new musical features for the computational model. To our knowledge, this was the first attempt to analyze the musical features of perceived immanent accents starting from empirical data and to model these features computationally. In the future, we plan to complete the music analysis of all the melodies, to refine the list of musical features, and to implement them in a future computational model.

In the modeling, the SVR method yielded the best results (*R*^2^ = 66%) for an optimized group of 15 participants, and this group was therefore used for investigating the influence of styles and participants on the modeling results. Modest variations due to the style were found, but the Post-tonal group differed from the others in that it was more influenced by the Pitch contour and Tempo features and less influenced by the Meter features. The individual modeling of each participant revealed large individual differences, indicating that the participants used the feature groups in different ways. We think that this aspect should be considered to a greater extent in future studies.

In the future, we will incorporate the hereby proposed immanent accent model into the KTH performance rule system (Friberg et al., 2000). Such a relationship and translation were realized in the earlier preliminary accent model described in Friberg and Bisesi (2014). In such an implementation, it would be natural to combine immanent and performed accents, e.g., by mapping the accent weight into changes of sound level or timing in the performance.

In the light of these results, we believe that our approach consisting of the close integration of computation, musicology, music analysis, and empirical methods to be very productive and one that should be pursued in future studies.

## Ethics Statement

This study was carried out in accordance with the recommendations of the Central Ethical Review Board in Sweden and according to Swedish law concerning the Ethical Review Act (2003:460). According to this law, the current experiment did not require an ethics approval. All participants gave written informed consent in accordance with the Declaration of Helsinki.

## Author Contributions

AF conceived the original idea, ran the computational modeling and wrote the first draft of the manuscript. EB performed the listening experiment, including stimuli preparation and recording, interface design and user analysis, wrote the corresponding parts in the manuscript, and contributed to the manuscript writing and editing. ARA and MB selected the music corpus, did the manual music analysis, and wrote the corresponding parts in the manuscript. All authors contributed to defining the methodology, the experimental protocol, and participated in the final proofreading.

## Conflict of Interest Statement

The authors declare that the research was conducted in the absence of any commercial or financial relationships that could be construed as a potential conflict of interest.

## Footnotes

1 The choice of terminology was non-trivial since we wanted to avoid the term “accent,” which would be confusing since there is a natural association to performed accent. “Prominence” could be another choice. However, we had many Swedish participants and a direct translation of “prominent” to Swedish does not correspond at all to what we aim for. Thus, it might have been more difficult to explain the experiment (the instructions were in English).
