# Supplementary material for: Probing the Underlying Principles of Perceived Immanent Accents Using a Modeling Approach
Source: Front Psychol. 2019 Jun 7;10:1024. doi: 10.3389/fpsyg.2019.01024 (PMC6566653; doi:10.3389/fpsyg.2019.01024)
Supplement: Supplementary file 1 [file Data_Sheet_1.pdf]

Supplementary material to Friberg, A., Bisesi, E., Addessi, A.R. & Baroni, M. (2019). Probing the underlying principles of perceived immanent accents using a modeling approach. *Frontiers in Psychology*, doi: 10.3389/fpsyg.2019.01024

1 Supplementary Material

1. Music material

Table S1. List of all of the melodies used in the experiment. B=Baroque, R=Romantic, PT=Post-tonal, I=Instrumental, V=Vocal.

Piece #	Style	Genre	Composer/Piece/Bars
1	B	I	VITALI, *Toccata* for solo cello (1-11)
2	B	I	MARINI, *L'Albana,* sinfonia breve (12-21)
3	B	I	COUPERIN, *L'Angélique* for harpsichord, 2nd band (1-19)
4	B	I	ALBINONI, *Sonata prima a 5* (from *Concerti e Sinfonie* Op. 2) (5-11, vl. I)
5	B	I	TORELLI, *Concerto* in E minor, Op. 8 No. 9 (1-14)
6	B	V	CARISSIMI, Cantata, *È bello l'ardire* (1-9)
7	B	V	RAMEAU, *Hyppolite et Aricie*, "Cruelle mère des amours" (28-38)
8	B	V	BONONCINI, *Il trionfo di Camilla regina dei Volsci,* Aria of Camilla, "Mi lusingo"
9	B	V	CHARPENTIER, *Judicium Salomonis* No. 5, Aria of Salomone (209-216)
10	B	V	MARCELLO, Arianna, Act I, *Ebbre bessaridi*
11	R	I	MENDELSSOHN, *Lieder ohne Worte*, Op. 19 No. 4 (9-17)
12	R	I	ROSSINI, *Guillaume Tell, Symphonie* (No. 7, Andante, from mark 8 to mark 9)
13	R	I	LISZT, *S. Francis of Assisi preaching to the birds* (52-63)
14	R	I	ČAJKOVSKIJ, *Symphony No. 6,* Allegretto con grazia (9-15 flutes +17-25 violins)
15	R	I	BERLIOZ, *Symphonie fantastique* III, *Scène aux champs* (French horn) (10-16)
16	R	V	BIZET, *Carmen,* Act III, "Recommence vingt fois" (5-15)
17	R	V	WAGNER, *Parsifal*, Act II, duet Kundry-Parsifal (verse 787: "die Wunde!") (2-16)
18	R	V	SCHUBERT, *Winterreise* No. 18, "Der stürmische Morgen" (3-18)
19	R	V	BELLINI, *I Capuleti e i Montecchi*, Act I, Scene IV, "Oh quante volte oh quante" (3-10)
20	R	V	SCHUMANN, *Dichterliebe* No. 6, "Im Rhein" (4-15)
21	PT	I	MADERNA, *Grande Aulodia* (oboe d'amore, from Langsam to the Corona)
22	PT	I	BERG, *Lyrische Suite,* II, Andante amoroso (1-8)
23	PT	I	WEILL, *Mahagonny,* Introduction (solo bassoon)
24	PT	I	SCELSI, *Quattro pezzi per tromba sola,* II
25	PT	I	BARTÓK, *Quartet No. 5,* 1st Mvt., vl. I
26	PT	V	STRAVINSKIJ, *Oedipus Rex,* Profezia di Tiresia, "Deus regem accusat" (from mark 79 to mark 83)
27	PT	V	BERIO, *Laborintus II* (Finale: "Ma seguimi, ma vedi i bambini", Voice 1[a])
28	PT	V	SZYMANOVSKI, *Op. 54,* "Sleep now" (9-17)
29	PT	V	NONO, *Il canto sospeso*, No. 5 (288-297)
30	PT	V	MESSIAEN, *Chants de terre et de ciel* (in Petrucci: from p. 14 - *joue à sautemouton,* to p. 15 - *malonlanlaine ma*)
31	B	I	SAULI, *Partita II for mandolin* (1-10)
32	B	I	CORELLI, *Sonate* for violin Op. 5 No. 7 (1-12)
33	B	I	SCARLATTI, *Sonate* for keyboard K 89 (1-11)
34	B	I	VIVALDI, *Concerto RV 425* in C major for mandolin, strings and continuum, 1st Mvt. (22-39)

35	B	I	BARBELLA, *Sonate Gimo 18* for 2 mandolins and bass, 2nd Mvt. (mandolin 1, without refrains)
36	B	V	PURCELL, *Dido and Aeneas*, Act III No. 38, Dido's Aria "When I'm laid in earth" (6-16)
37	B	V	HÄNDEL, *Rinaldo*, Aria "Cor'ingrato" (1-14)
38	B	V	BACH, Choral "Gottes Sohn ist kommen"
39	B	V	PERGOLESI, "Luce degli occhi miei" (1-15)
40	B	V	CACCINI, "Ch'amor sia nudo" (1-18)
41	R	I	BEETHOVEN, *String Quartet,* Op. 131 No. 14, III Mvt, (254-277)
42	R	I	BRAHMS, *Intermezzo,* Op. 117 No. 1, inner melody – rh (1-8)
43	R	I	GRIEG, *Sonate for violin and piano* Op. 45*,* 1st Mvt. (4-15)
44	R	I	WIECK-SCHUMANN, *Soirées Musicales*, No. 2, Nocturne (2-24)
45	R	I	MASCAGNI, *Cavalleria Rusticana,* Intermezzo sinfonico, vl. I (1- 10)
46	R	V	MAHLER, *Simphony No. 4,* IV Mvt., "Sehr behaglich", voice (13-25)
47	R	V	MASSENET, *Dèpart* (2-10)
48	R	V	ALBÉNIZ, *Rimas de Bécquer* (1-8)
48	R	V	DONIZETTI, *Linda di Chamounix, "*O luce di quest'anima" (10-25)
50	R	V	NORMAN, *Svar* (1-18)
51	PT	I	KURTÁG, *String Quartet* Op. 1*,* 5th Mvt., vl II (2-11)
52	PT	I	MILHAUD, *Quartet No. 7,* 1st Mvt., vl. I (1-14)
53	PT	I	GRISEY, *Vortex Temporum* for piano and six instruments, flute (1-25)
54	PT	I	TAKEMITSU, *All in Twilight for Guitar* (1-15)
55	PT	I	BOULEZ, *Mémoriale* (explosante-fixe… Originel) for solo flute and eight instruments, flute, (1-15)
56	PT	V	SCHOENBERG, *Pierrot Lunaire* Op. 21, II, "Colombine", voice (1-12)
57	PT	V	DALLAPICCOLA, *Cinque frammenti di Saffo* for voice and chamber orchestra, voice (3-7)
58	PT	V	VARÈSE, *Offrandes* for soprano and chamber orchestra, II, "La croix du sud", voice (8-20)
59	PT	V	SCIARRINO, *Vanitas* for voice, cello and piano, II, "Marea di rose", voice (7-17)
60	PT	V	WEBERN, *Canon,* Op. 16 No. 2 for voice and clarinet, voice (2-7)

2. Detailed questionnaire results

The first part of the questionnaire concerned the musical expertise and is reported in the section "Listening Experiment". The second part of the questionnaire was dedicated to familiarity with the styles. After each round, we asked the participants whether they recognized if any of the melodies were from Medieval, Renaissance, Baroque, Classical, Romantic, and Modern and/or contemporary styles of music. Overall, the Baroque style was recognized rather well (72% of cases), while the Romantic style was recognized rather poorly (37% of cases), and the Post-tonal style was rather easily distinguished (82% of cases). Despite the uniform distribution of our repertoire among the three styles (Baroque, Romantic and Post-tonal), the presence of many occurrences of the other three alternatives (39 Classical, 9 Medieval and 5 Renaissance occurrences within the two rounds) revealed a significant degree of confusion, possibly between the Romantic and the Rlassical style.

After each melody, participants were also asked to indicate whether they recognized it. The result was that the participants rarely recognized the melodies. In total, 22 melodies were recognized by at least one participant (ranging from 1 to 7), and 19 participants recognized at least one of the melodies (ranging from 1 to 7).

In the third part of our questionnaire, we explored the criteria adopted to rate the important notes in the melodies. Eleven options were proposed (rhythm/meter, melody, harmony, pitch, loudness, duration, articulation, timbre/sonority, phrasing, pauses/silences, other) and participants were asked to select one or more of these. They also had the possibility of suggesting alternative principles. As we can see in Figure S1, the main focus was placed on rhythm/meter and melody. To a lesser extent, phrasing, duration, harmony, pauses/silences were also considered. Among the alternative criteria adopted for selection, there were the comparison between subsequent elements in a melody (e.g., the size of melodic leaps), and the comparison with other well-known melodies.

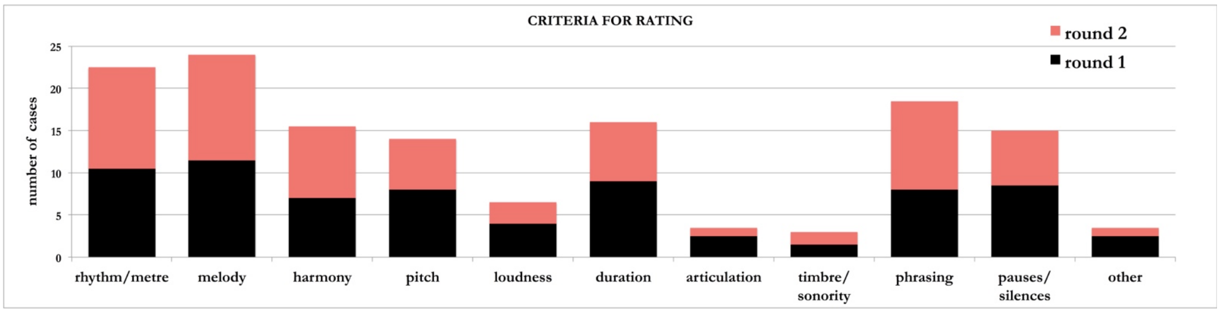

Figure S1. Criteria reported by the participants for rating the melodies.

The questionnaire concluded by asking whether there was any difficulty associated with the task. Eighteen participants reported awkwardness during the first round, 12 of whom confirmed their statement after the second round. Thus, several participants became more used to the task in the second round. Reported difficulties were: troublesome to select among the [too] many aspects that may contribute to make a note important in a melody, given also that they often contradict each other; too many examples; too many notes in some examples; in some melodies, participants reported problems in detecting beats or dealing with pauses, as well as making their subjective perception of meter fit with that of the melodic line; some

melodies were judged as too fast; it was uneasy to be consistent along a piece, or between subsequent listenings of the same piece; sometimes it was hard to choose among the criteria for selection in the questionnaire; it was hard to maintain the concentration for the whole duration of the task; some hardship with "boring" pieces, mostly Post-tonal (as "… there was no point of interest, or all the notes were equally interesting"). Representatively, one participant reported: "I did not exactly understand the question (i.e. rate/select the most important notes), since there might be notes that are "musically important" but do not draw any attention, and vice versa. Also, I believe that in some cases I selected [too] many notes, or rather phrases, as [my] attention was drawn to a particular sequence of notes rather that to an isolated note. Also, there were some melodies in which no note caught my attention."

3. Scores analyzed in the section "Results of Music Analysis of the "Strong" Accents, With All Participants (Mean)"

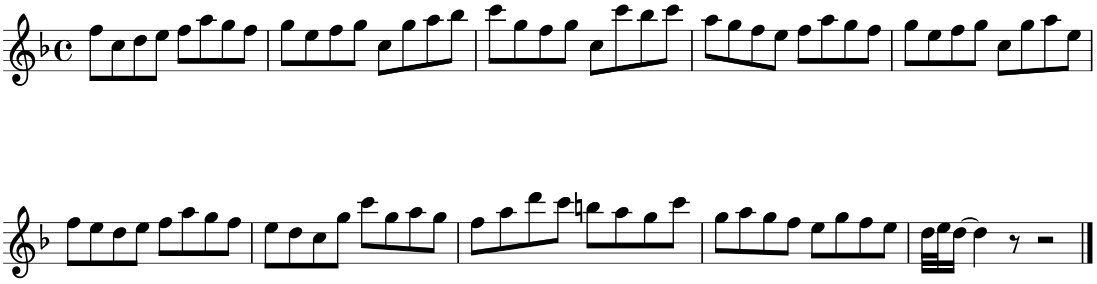

Figure S2. Melody No. 31 by Sauli.

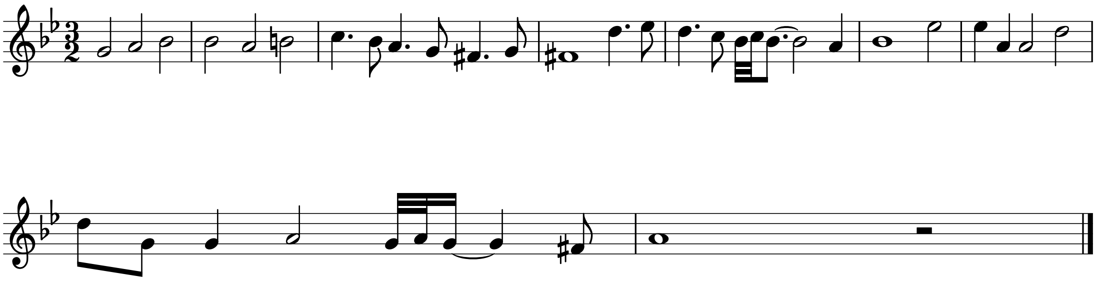

Figure S3. Melody No. 36 by Purcell.

3. Definition of the metrical levels

Table S2. Selection of the metrical levels beat0-beat3 used in the Meter features presented in section "Simple Phrasing", for the different meters notated in the music database. Note that beat1 corresponds to the pulse or tactus level. For many of the compound meters, such as 5/4, it was not possible to make a generic subdivision on the half measure level (beat2). In these cases, beat2 was chosen as the same as beat3.

Meter	beat0	beat1	beat2	beat3
Simple				
4/4	1/8	1/4	2/4	4/4
2/4	1/8	1/4	2/4	4/4
8/4	1/8	1/4	2/4	4/4
8/16	1/8	1/4	2/4	4/4
2/2	1/4	1/2	2/2	4/2
4/2	1/4	1/2	2/2	4/2
3, 6, 9, and 12				
3/4	1/8	1/4	3/4	6/4
3/2	1/4	1/2	3/2	6/2
6/4	1/8	1/4	3/4	6/4
3/8	1/8	3/8	6/8	12/4
6/8	1/8	3/8	6/8	12/8
6/16	3/16	3/16	6/16	12/16
9/8	1/8	3/8	9/8	18/8
9/16	3/16	3/16	9/16	18/16
12/8	1/8	3/8	6/8	12/8
12/16	1/16	3/16	6/16	12/16
Compound				
5/4	1/8	1/4	5/4	5/4
5/16	5/16	5/16	10/16	20/16
7/16	7/16	7/16	7/16	14/16
10/16	10/16	10/16	10/16	20/16
11/16	11/16	11/16	11/16	22/16
13/16	2/16	4/16	13/16	13/16
14/16	7/16	7/16	7/16	14/16
15/16	1/16	3/16	15/16	15/16
17/16	2/16	4/16	17/16	17/16
19/16	19/16	19/16	19/16	19/16
Special				
25/4	1/8	1/4	2/4	4/4
34/4	1/8	1/4	2/4	4/4
